# Fecal Microbiota Transplant in Severe and Non-Severe *Clostridioides difficile* Infection. Is There a Role of FMT in Primary Severe CDI?

**DOI:** 10.3390/jcm10245822

**Published:** 2021-12-13

**Authors:** Daniel Popa, Bogdan Neamtu, Manuela Mihalache, Adrian Boicean, Adela Banciu, Daniel Dumitru Banciu, Doru Florian Cornel Moga, Victoria Birlutiu

**Affiliations:** 1 Faculty of Medicine, Lucian Blaga University of Sibiu, 550169 Sibiu, Romania; danieliulian.popa@ulbsibiu.ro (D.P.); manuela.mihalache@ulbsibiu.ro (M.M.); adrian.boicean@ulbsibiu.ro (A.B.); cornel.moga@ulbsibiu.ro (D.F.C.M.); victoria.birlutiu@ulbsibiu.ro (V.B.); 2 Dr. Alexandru Augustin Military Hospital of Sibiu, 550024 Sibiu, Romania; 3 Polisano Clinic Sibiu, 550253 Sibiu, Romania; 4 Pediatric Research Department, Pediatric Clinical Hospital Sibiu, 550166 Sibiu, Romania; 5 County Clinical Emergency Hospital of Sibiu, 550245 Sibiu, Romania; 6 Department of Bioengineering and Biotechnology, Faculty of Medical Engineering, Polytechnic University of Bucharest, 011061 Bucharest, Romania; adela.banciu79@gmail.com (A.B.); danieldumitrubanciu@gmail.com (D.D.B.)

**Keywords:** *Clostridioides difficile*, primary infection, fecal microbiota transplant, recurrence

## Abstract

Background: Faecal microbiota transplant (FMT) is a highly effective therapy for recurrent *Clostridioides difficile* infection (rCDI) with cure rates ranging between 85 and 92%. The FMT role for primary *Clostridioides difficile* infection (CDI) has yet to be settled because of limited data and small-sample studies presented in the current literature. Our study goals were to report the risk factors and the risk of recurrence after FMT for each CDI episode (first, second, and third) and to explore if there is a role of FMT in primary severe CDI. Methods: We conducted a retrospective study to analyze the clinical characteristics and the outcomes of 96 FMT patients with a prior 10 day course of antibiotic treatment in the medical records, of which 71 patients with recurrent CDI and 25 patients with a primary CDI. Results: The overall primary cure rate in our study was 88.5% and the primary cure rate for the severe forms was 85.7%. The data analysis revealed 5.25%, 15.15%, and 27.3% FMT recurrence rates for primary, secondary, and tertiary severe CDI. The risk of recurrence was significantly associated with FMT after the second and the third CDI severe episodes (*p* < 0.05), but not with FMT after the first severe CDI episode. Conclusions: This study brings new data in supporting the FMT role in CDI treatment, including the primary severe CDI, however, further prospective and controlled studies on larger cohorts should be performed in this respect.

## 1. Introduction

In recent years, CDI has become an actual social problem due to the increase in its incidence and severity. *Clostridioides difficile* (CD) is an anaerobic, spore-forming, Gram-positive bacteria. It is an opportunistic pathogen in gut microbiome dysbiosis, spreading its toxins on the colonic mucosa after antibiotic exposure. Although antibiotics are effective in CDI treatment, the main drawback is dysbiosis, with a consequence on gut-barrier protection from other pathogens. The affected microbiota further impairs the immune-mediated response. The pathogenic immune responses involve both innate and adaptative immunity. Innate immune responses type 1 and type 2 are of paramount importance in CDI. Innate immune response type 1 with the innate lymphoid cells expressing (IFN)-γ, IL-12,18,22, and innate immune response type 2 with eosinophils attracted by IL-5,24,33 cytokines, are crucial to CDI recovery and are modulated by microbiota. The adaptative immune responses with Th17 cells as key players also have an important role in CDI colitis. Microbiota’s signals modulate the interplay between Th17 cells, the innate immune cells type 3, and the epithelial cells towards an effective immune response. Studies on germ-free laboratory animals have highlighted the microbiota importance linked with each of the aforementioned pathogenic responses. FMT alleviated CDI colitis recovery. The presented mechanisms might explain risk factors such as antibiotic dysbiosis, age, or immunodeficiencies that alter the microbiome composition and/or the immune response efficiency. These risk factors increase the odds for CDI relapse and for CDI severe forms [1]. 

CDI, antibiotic-associated colitis, represents the most common cause of diarrheic disease associated with public health services, accountable for an important morbidity and mortality rate. After a first CDI episode, 11–25% of patients will relapse within the first 30 days after the completion of therapy [2,3,4,5,6]. Following the first relapsing episode, 46.2% of the patients will have a second relapsing episode [7]. Furthermore, the risk of recurrence continues to rise, reaching 50–60% after the third episode [8]. 

FMT proved to be an effective treatment with a higher than 90% cure rate for the rCDI patients when randomized controlled trials (RCTs) were published [9,10,11,12]. Afterward, systematic reviews and meta-analyses have confirmed a cure rate ranging between 85% and 92%, with lower rates in RCTs compared with nonrandomized trials [13,14,15].

Currently, the FMT is included in the European CDI treatment guidelines for multiple recurrences [16], mild or severe rCDI, and refractory CDI [17], while in the American CDI treatment guidelines, after the second or subsequent rCDI [18].

The literature, however, is scarce regarding the FMT role for the first CDI episode. Only a small number of patients were reported in previous studies [14,19,20,21]. We included in our retrospective study patients with a prior 10 day-course antibiotic treatment in the medical records. These patients were at first, second, or third CDI episodes with both severe and non-severe CDI forms. Our main goal was to determine the risk of recurrence after FMT for each episode and to establish if there is a role of FMT in primary severe CDI. We analyzed the FMT primary success rate (overall or after a severe form), the secondary success rate, the risk factors for post-FMT recurrence, and the median days of the recurrence.

## 2. Materials and Methods

### 2.1. Study Design

**Figure 1:**
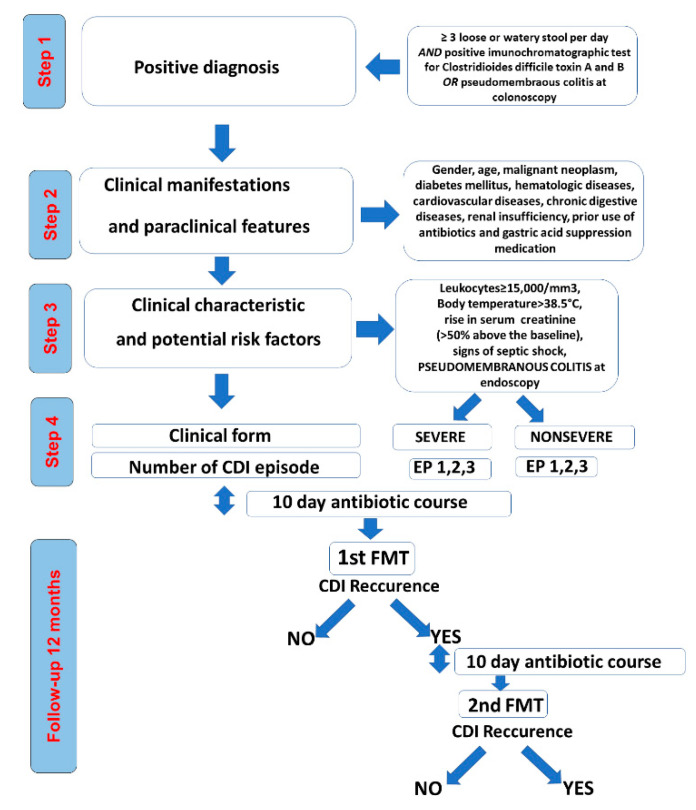
Study design.

In our retrospective study, we examined the clinical characteristics and outcomes for 96 CDI patients with FMT from January 2015 to July 2019 (Figure 1).

The research was conducted in Sibiu County Clinical Emergency Hospital (Gastroenterology and Infection Disease Departments) and Sibiu Polisano Clinic (Gastroenterology Department), Romania. All the FMT procedures were undertaken in these two major medical institutions in the county. The study design was conceived according to the Helsinki Declaration Ethical principles and was approved by the Institutional Ethical Committee.

### 2.2. Study Population

We included in the study only the patients aged >18 years, with a first FMT performed after a 10 day antibiotic course for a CDI episode, irrespective of the CDI episode number or severity form. Exclusion criteria were previous FMT, other FMT routes than colonoscopic method or incomplete follow-up (less than 12 months post-FMT).

### 2.3. Patients’ Demographic and Clinical Characteristics

The data collection was retrieved from the electronic medical records available upon request according to the institutional procedures for clinical retrospective studies. Demographic characteristics included age and gender. We analyzed all the medical records, including antibiotherapy 2 months before CDI, and we examined the existing data on clinical outcomes 12 months after FMT [22].

Clinical characteristics included the following: (1) potential risk factors—comorbidities (malignant neoplasms, diabetes mellitus, hematologic diseases, cardiovascular diseases, chronic digestive diseases, renal insufficiency), prior use of antibiotics and gastric acid-suppression medication; (2) clinical manifestations and paraclinical features—leukocytes, body temperature, serum creatinine, signs of septic shock, pseudomembranous colitis at endoscopy; (3) treatment and outcome—CDI episode number, at which CDI episode FMT was performed, recurrence after FMT, the number of days of CDI recurrence, severe forms, non-severe forms [22].

### 2.4. Outcomes

This study aimed to assess primarily the risk of recurrence post-FMT procedure performed after a complete 10 day course of antibiotics and the resolution of the symptoms after a primary severe CDI episode. As secondary outcomes, we focused on: (i) the overall primary and secondary FMT success rates, (ii) the general FMT success rate after a severe form; (iii) the risk factors for post-FMT recurrence, (iv) the median days of recurrence, and (v) the recurrence risk for the post-FMT procedure employed after a second and a third severe CDI episode.

### 2.5. Definitions

CDI was defined as diarrhea (≥3 loose or watery stools per day, corresponding to Bristol stool chart types 5–7, for the last 48 h) and either the positive immunochromatographic test for *Clostridioides difficile* toxin A and B or the colonoscopic findings demonstrating pseudomembranous colitis [16]. The severe CDI form was defined using at least one of the proposed criteria by the European Society of Clinical Microbiology and Infectious Diseases (ESCMID) [16] and the Infectious Diseases Society of America (IDSA)/Society for Healthcare Epidemiology of America (SHEA) [18]: leukocytosis ≥ 15,000/mm^3^, body temperature > 38.5 °C, rise in serum creatinine (>50% above the baseline), signs of septic shock, pseudomembranous colitis at endoscopy.

The primary nonrecurring CDI episode was defined as the first CDI episode while the recurring CDI episodes were referred as the second and the third CDI episodes. According to each case situation, the first FMT was performed after a complete 10 day course of antibiotics at the first, second, or third CDI episode. FMT failure was defined as recurrent diarrhea confirmed by a positive immunochromatographic test for *Clostridioides difficile* toxin A and B or pseudomembranous colitis at colonoscopy. A second FMT was performed after the first FMT failure (Figure 1). For these patients with a second FMT we used the same colonoscopic method for FMT delivery, after a 10 day course of antibiotics, but a different donor.

*Faecal microbiota transplant (FMT)’s* timing was chosen 24–48 h after a 10 day course of antibiotic, as suggested in other literature reports [23,24,25,26]. Irrespective of CDI episode number, the patients were selected for FMT procedure either with severe CDI forms or with non-severe CDI forms. In non-severe CDI forms, the following categories were considered: patients aged > 65years with comorbidities, patients aged < 65years of age with chronic digestive diseases or with other comorbidities or patients, irrespective of the age, who chose the FMT willingly, being aware of the relapse risk just after the 10 day course of antibiotic treatment.

*Donor screening:* the donors were chosen from the family members or close friends without auto-immune diseases or other chronic conditions, without the use of antibiotics within the last 3 months. Donor feces were screened for ova and parasites, Giardia antigen, stool culture, and sensitivity test for enteropathogenic bacteria (Salmonella, Shigella, Escherichia Coli, Yersinia Enterocolitica, Campylobacter), immunochromatographic test for *Clostridioides difficile* toxin A and B. Donor serum was screened for antibodies against HIV-1, HIV-2, Hepatitis A, B, C, and VDRL.

*Patient preparation:* a 4 L bowel lavage with polyethylene glycol (PEG) was administered the evening prior to the transplant.

*Feces preparation*: At least 30 g of fresh donated feces (<6 h) was mixed with 500 mL of normal saline (0.9%) in a single-use, tightly closed sterile container and vigorously stirred; the resulting mixture was filtered in another single-use sterile container through multiple gauze layers.

*Instillation technique*: a colonoscopy, under conscious sedation, was performed trying to reach the distal segment possible (preferable terminal ileum); with a large volume syringe (50 mL), the suspension was delivered through the working channel in the terminal ileum and/or the right colon—2/3 of the total volume and the rest during withdrawal.

*Post-transplant*: two tablets of loperamide were administered immediately after the procedure and one after 6 h, with bed rest as long as possible.

The patients were monitored periodically by telephone for 12 months after FMT.

### 2.6. Data Analysis

Data are presented as percentages and medians. The potential risk factors for the outcomes consisted of age (computed as a numerical variable in years) and the following as categorical variables: (1) gender, (2) comorbidities categories (malignant neoplasms, diabetes mellitus, hematological diseases, cardiovascular diseases, chronic digestive diseases, renal insufficiency), (3) clinical manifestations and paraclinical features (leukocytes, body temperature, serum creatinine, signs of septic shock, pseudomembranous colitis at endoscopy), (4) antibiotherapy or gastric acid-suppression medication prior to CDI, antibiotherapy classes (Aminopenicillins ± Beta-lactamase inhibitor, Cephalosporins, Carbapenems, Fluoroquinolones, and Clindamycin). Both the primary and the secondary outcomes were interpreted as categorical variables: (i) the post-FMT procedure recurrence after a primary severe CDI episode; (ii) the primary overall FMT success, (iii) the recurrence post-FMT procedure employed after a second and a third severe CDI episode, (iv) the secondary overall success, (v) the post-FMT recurrence after a non-severe first, second, or third CDI episode.

We applied Fisher’s exact test for the categorical variables and Mann–Whitney test for continuous variables, respectively [27,28,29]. The *p*-value < 0.05 was considered statistically significant. However, we also considered “trending toward clinical studies” to be clinically relevant and appropriate in our small-sample retrospective study using the relaxed value of the α level to 0.10 as suggested by S. Thiese et al. [27].

The statistical analyses were conducted in SPSS software v.28.

## 3. Results

### 3.1. Study Population and CDI Risk Factors

This study included 96 CDI patients with a prior 10 day-course antibiotic treatment in the medical records. There were 67 female patients (69.8%), the median age was 68.5 (range 20–89 years) and 58 patients (60.4%) were aged >65 years. Sixteen patients (16.7%) did not have comorbidities. Cardio-vascular diseases were the most common comorbidity found in 58 patients (60.4%) and 46 patients (47.9%) had more than two comorbidities. For 30 patients (31.3%), we did not find any previous antibiotherapy administration record before the onset of the first infectious episode. Cephalosporins were the most common antibiotics administered for 41 patients (42.7%), in 23 patients (24%), we documented antibiotic associations, while in 31 patients (32.3%) the treatment included the association of an antisecretory gastric drug. The demographic and clinical characteristics are presented in Table 1.

Seventy-one patients had recurrent CDI, while 25 patients were documented with a primary CDI. Sixty-three patients (65.6%) developed the severe form of the disease, while 33 patients (34.4%) developed a non-severe CDI form previous to the FMT. Among the patients with the severe form, 26 patients (41.3%) had only one factor of severity, while 37 patients (58.7%) had ≥2 severity factors. From 58 patients aged >65 years, 41 patients (70.1%) developed a severe form. However, the age >65 years was not a risk factor in this respect (*p* = 0.197). On the same note, there was no association between the severe-form development and the comorbidities presence even in the presence of ≥2 comorbidities documented for the respective cases. The overall antibiotic treatment administered before the CDI onset was not associated with the risk of developing a severe form. Nevertheless, the analysis on the antibiotic-type subgroups revealed that the carbapenems treatment and gastric antisecretory medication prior to the onset of the infectious episode were associated with the severe form development (*p* = 0.067 and *p* = 0.000, respectively). 

### 3.2. Fecal Microbiota Transplant

All 96 patients received a first FMT procedure, of which only 11 had a second FMT.

**Figure 2:**
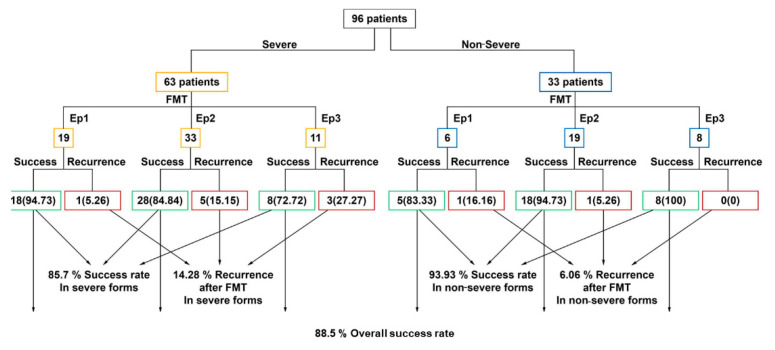
FMT success and recurrence rates, numbers (percentages).

The cases distribution based on FMT procedure performed at first, second, or the third CDI episode was the following: (1) Out of 96 patients, in 25 patients (26%) FMT was performed at the first infectious episode, (2) in 52 patients (54%), at the second infectious episode, and (3) in 19 patients (20%) at the third infectious episode (Figure 2).

Only one patient with a severe form at the second infectious episode deceased, but from causes unrelated to the FMT.

The primary success rate in patients with a first FMT was 88.5% (85 patients did not have a CDI relapse), regardless of severity form. The FMT success rate per episode was 92% at the first episode, 88.4% at the second, and 84.2% at the third CDI episode. However, 11 patients (11.5%) with a first FMT showed a CDI relapse (Figure 2).

### 3.3. Recurrence after FMT

Out of the 11 patients with a post-FMT relapse, we recorded the following relapse rates: (i) two patients with the FMT made after the first CDI (8% relapse rate); (ii) six patients with the FMT performed after the second CDI (11.5% relapse rate); (iii) three patients with the FMT after the third CDI (15.8% relapse rate) (Figure 2).

The median number of post-FMT relapse days was 10.5 (range 1–190). The risk of relapse post-FMT was not statistically correlated with the patients’ age, the presence of comorbidities, the antibiotic use/antibiotic types, or the use of gastric antisecretory medication prior to the first CDI episode.

The initial FMT success rate in severe forms was 85.7%. However, out of the 11 relapsing patients, nine experienced the relapse event after a severe form of the disease: (i) one patient with the severe form with FMT at first CDI (a relapse rate of 5.26%); five patients with severe form relapsed after FMT performed at the second CDI (a relapse rate of 15.15%); three patients relapsed after the FMT completed at the third infectious episode had a severe form of the disease, representing a relapse rate of 27.3%.

The overall post-FMT relapsing risk was not statistically correlated with the severe forms (*p* = 0.229) or severe forms associating only one severity criterium (*p* = 0.480). However, there was a correlation with the severe forms associating with two or more severity criteria (*p* = 0.069).

Further analysis on the severe-form patients’ data revealed no recurrence risk with the FMT performed after the first infectious episode (*p* = 0.358). Nevertheless, there was a statistically significant risk for the relapsing event correlated with further infectious episodes in which the FMT was performed: (i) the 2nd infectious episode (*p* = 0.017); (ii) the 3rd infectious episode (*p* = 0.002) (Table 2).

In the non-severe-CDI-form patients category (33 patients), a 93.93% overall primary success rate was noticed after the first FMT and 6.06% recurrence rate: (1) only one out of six patients (16.6%) relapsed after the FMT performed for primary CDI and only 1 out of 19 patients (5.26%) relapsed after the FMT performed for the second CDI episode. None of the patients with a non-severe form at the third CDI episode relapsed after FMT (Figure 2).

The patients with a second FMT, eight with severe form and three with a non-severe form, did not present another CDI relapse during their follow-up, resulting in a secondary success rate of 100%.

## 4. Discussion

This retrospective study highlights the FMT outcomes in 96 patients at different CDI episodes. We included the patients at the first, second, and third CDI episode, to determine the risk factors and the risk of recurrence after FMT for each episode and to explore if there is a role of FMT for primary severe CDI.

When we performed the first FMT, there was no consensus on donor screening, pretransplant preparation, the amount of feces turned into suspension to be administered, posttransplant conduct or the administration methodology. We chose colonoscopy as the administration method, this route being associated with higher efficiency compared to the upper route [13]. Our protocol was established based on the recommendations at that time [23,24,25,26]. Those recommendations were later proposed in the European Consensus on FMT [17].

### 4.1. CDI Forms, the Risk Factor Association, and FMT Success Rates

Our reports partially align with other authors’ data regarding the association of the acknowledged risk factors and CDI forms. In this study, age was not a favoring factor for severe form development, even in patients aged >65 years, compared to other studies results [30]. However, patients aged >65 years without severe-form criteria, with comorbidities, hospitalized in ICU, or with immunodeficiencies should be considered as having a severe form [31].

There was a female gender predominance in the study cohort, similar to other studies’ reports, but this association is not entirely understood, requiring further research [32,33]. Some literature reports highlighted that the overall antibiotic treatment preceding the CDI episode was not associated with the risk of developing a severe form, regardless of the antibiotic class [30]. However, other research papers revealed a risk for the severe form being associated with the carbapenems treatment [34]. Our study is in line with the latter. The risk of superinfection is increased to approximately 70% for carbapenems compared with non-carbapenems antibiotics. This higher rate could be explained by the relatively broader spectrum of activity compared to other antibiotics, leading to lower microbiota diversity, dysbiosis, and changes in the metabolic pathways [35].

Furthermore, we agree with the current literature reports on gastric acid suppression related to the development of severe CDI forms [36,37]. In these patients, failure to inactivate the vegetative form of CD in a less acidic medium could explain the exacerbation or prolongation of CDI [38].

We could not document any association between the post-FMT relapse risk and risk factors such as age, antibiotics use, antisecretory medication, or comorbidities’ presence. The literature is controversial regarding the association between post-FMT relapse risk and the aforementioned risk factors. For example, in a recent meta-analysis, Tariq et al. highlighted non-CDI antibiotic medication as a post-FMT relapse risk factor [39], contrary to several other studies [22,40]. We agree with the latter. On the other hand, regarding the relapse risk and the severe/complicated CDI forms, both Tariq et al., in their meta-analysis [39], and Fischer et al., in a larger cohort study on 328 patients [22], reported an association in this respect [22,39]. Our study categorized the cases into severe forms presenting one severity criterium and two or more severity criteria, with a post-FMT relapse risk only in the latter. We believe that classifying the patients in terms of one severity criterium and two or more severity criteria forms might offer a novel study design approach that could be further validated in future multicentric randomized controlled studies on larger cohorts.

Our study’s overall FMT success rate was 88.5%, comparable with data presented in other studies, systematic reviews, and meta-analysis [9,10,11,12,13,14,15]. In this study, the success rate after the first FMT for severe forms irrespective of the CDI episode number was 85.7%, and comparable to other studies’ data (79.6–91%) [22,33,41], to finally reach at 100% success rate after a second FMT [22,42].

### 4.2. FMT Role and Success Rates in Primary Nonrecurring Severe and Non-Severe CDI Forms

When it comes to FMT’s role in primary, nonrecurring severe and non-severe CDI forms, the results should be interpreted with caution based on the research design. We report 25 patients with primary CDI FMT, 19 with a severe and 6 with a non-severe form. Out of six patients with a non-severe primary CDI form, three patients were aged >65 years with comorbidities, two patients aged <65 years with chronic digestive diseases, and one patient aged <65 years who chose the FMT willingly. Bauer et al. recommended that patients aged >65 years with comorbidities and without severe-form criteria should be considered as having a severe form [31], and according to Goodhand et al., inflammatory bowel diseases seem to be associated with a severe CDI prognosis [43]. We recorded a single case, a female patient with inflammatory bowel disease with an infectious relapse after the FMT employed for a non-severe primary CDI form. According to Tariq et al.’s meta-analysis, inflammatory bowel disease is associated with a post-FMT relapse risk [13]. Another unexplainable relapse in a non-severe form was registered in a young patient without comorbidities after a second CDI episode.

The primary severe, nonrecurring CDI FMT success rate in our cohort was 94.7%. The literature mentioning the relapse rates after FMT in patients with primary, nonrecurring CDI is scarce. Zainah et al. included in their study six patients with severe primary CDI form, of which three relapsed after the first FMT [41]. Other published data on severe or complicated primary CDI cases referred to a general success rate but counting also the CDIs of recurring patients. Fischer et al. reported nine patients with severe primary CDIs, while Kelly et al. studied 15 patients with severe primary CDIs, moderate primary CDIs, and mild primary CDIs [14,42]. These studies, however, did not mention a specific primary CDI success rate. In a more recent paper, the authors presented a survey of 44 patients with FMT for primary CDI that completed a stool test 4–8 weeks after the treatment and had an impressive success rate of 98% CDI-negative patients [21]. This study brought new data for primary CDI FMT. Nevertheless, the authors did not separate the patients into non-severe and severe forms, which might explain a higher success rate. Notwithstanding, our results related to FMT in the primary severe and non-severe CDI forms (92%) are close to these reports.

### 4.3. FMT Relapse Rates after the First, Second, and Third CDI Episodes

Further on in our analysis, we noticed important differences regarding the post-FMT relapse rates. First, we noted a 5.25% post-FMT relapse rate in severe primary CDI, much lower than in Lagier et al.’s reports (37.5%) [19]. A possible explanation might be that Lagier and his colleagues [19] presented patients infected with *Clostridioides difficile* ribotype 027 during an outbreak in Marseille, a more virulent strain, associated with significantly higher morbidity, mortality, and related to more severe complications [44]. Moreover, after the first CDI episode, the relapse rates treated only with antibiotics mentioned in the literature are between 11 and 25% [2,3,4,5,6]. In our case, a lower relapse rate after FMT at first CDI episode is theoretically associated with lower costs related to hospitalization, treatment, and adverse events for these patients. In their study, Lagier and his colleagues recorded a fivefold decrease in mortality rate in the early FMT group versus the conventional-therapy group. Although their findings should be interpreted in epidemic settings with a hypervirulent ribotype 027, we should remember that early FMT may be beneficial in an epidemic setting with a high mortality rate [19].

Second, an important observation in our study refers to the recurrence rate in the severe form that was significantly associated with FMT performance at the second and the third CDI severe episode. However, we could not document this association with the FMT performance after the primary severe CDI episode. The post-FMT relapse rate increased with the infectious episodes, from 5.25% after severe primary CDI, to 15.15% and 27.3% after the second and third episodes of severe CDI, pleading for antibiotherapy an early FMT in the severe forms.

To resume, for the moment, there are insufficient data to make recommendations regarding FMT in the primary CDI [17]. To the best of our knowledge, we are reporting a higher number of patients with an FMT for primary severe CDI, a more extended follow-up period after FMT, and an original design dividing patients with the severe form according to the severity criteria number. However, our study has some limitations in terms of small sample size, the retrospective design with selection bias compared to randomized control trials, and absence of *Clostridioides difficile* ribotyping. Future randomized controlled trials in multiple clinical centers with larger datasets could further explore the FMT role for primary severe CDI and its role in severe-form categories based on severity-criteria combination.

## 5. Conclusions

This study brings new data to support the role of FMT with a very low recurrence rate in the primary severe CDI, and an increased recurrence rate with the number of CDI episodes. Prospective and controlled studies are warranted to determine the role of FMT in the primary CDI, in particular primary severe CDI, but until then we should bear in mind that FMT could be the right tool for the primary severe CDI or severe complicated CDI.

## Figures and Tables

**Table 1:** Demographic and clinical characteristics of the patients.

Number of Patients	96
**Age**	
Median, years (range)	68.5 (20–89)
20–39 years, *n* (%)	15 (15.6)
40–59 years, *n* (%)	13 (13.5)
60–79 years, *n* (%)	56 (58.3)
80–90 years, *n* (%)	12 (12.5)
>65 years, *n* (%)	58 (60.4)
**Gender**	
Female, *n* (%)	67 (69.8)
Male, *n* (%)	29 (30.2)
**Comorbidities**	
No, *n* (%)	16 (16.7)
Malignant neoplasm, *n* (%)	15 (15.6)
Diabetes mellitus, *n* (%)	13 (13.5)
Hematologic Diseases, *n* (%)	11 (11.5)
Cardiovascular diseases, *n* (%)	58 (60.4)
Chronic digestive diseases, *n* (%)	38 (39.6)
Renal insufficiency, *n* (%)	14 (14.6)
≥2, *n* (%)	46 (47.9)
**Antibiotics use prior to CDI**	
No, *n* (%)	30 (31.3)
Aminopenicillins ± beta lactamase−inhibitor, *n* (%)	14 (14.6)
Cephalosporins, *n* (%)	41 (42.7)
Fluorochinolone, *n* (%)	27 (28.1)
Carbapenems, *n* (%)	6 (6.3)
Clindamycin, *n* (%)	1 (1)
Associations, *n* (%)	23 (24)
**Acid-suppression medications**	
No, *n* (%)	65 (67.7)
Yes, *n* (%)	31(32.3)

**Table 2:** Recurrence risk in severe forms.

Severe Form of CDI	Total no FMT	No. of Recurrent CDI after FMT (%)	Fisher’s Exact Test
1st CDI episode	19	1 (5.26%)	0.358
2nd CDI episode	33	5 (15.15%)	0.017
3rd CDI episode	11	3 (27.3%)	0.002
Total	63	9	-

## Data Availability

The data presented in this study are available on request from the corresponding author. The data are not publicly available due to data protection legislation.
